# Lifestyle for a lifetime

**DOI:** 10.1007/s12471-015-0764-8

**Published:** 2015-11-03

**Authors:** E.E. van der Wall

**Affiliations:** Netherlands Society of Cardiology/Holland Heart House, Moreelsepark 1, 3511 EP Utrecht, The Netherlands

Current guidelines for prevention and treatment of cardiovascular disease (CVD) emphasise the importance of a healthy lifestyle. There is now sufficient evidence that a multitude of lifestyle factors play an important role in the occurrence of CVD. Most of these factors relate to lifestyle and are thus modifiable, such as tobacco smoking, lack of physical activity, dietary habits, elevated blood pressure, type 2 diabetes, and dyslipidaemias. Consequently there has to be continuous attention to lifestyle modification, both in primary and secondary prevention [1]. With respect to secondary prevention, cardiac rehabilitation has become increasingly important as has been underscored by the study by Achttien et al. [2] in this issue of the Netherlands Heart Journal. Before dwelling on their study, I would first like to stress the importance of lifestyle, its components and its behavioural aspects. Interestingly, the value of the individual lifestyle factors may change over time and new lifestyle factors appear on the horizon. In short communication the most recent observations (second half year 2015) and changes in the area of lifestyle will be addressed as well as some new lifestyle issues.

## Physical activity

- Short bouts of high-intensity exercise can reverse some early cardiac changes in patients with type 2 diabetes [3]. It was reported that high-intensity intermittent exercise significantly improves heart structure and function.
- Sitting is bad for children indicating that children who sit too much may face adult-sized health consequences [4]. After a single session of prolonged inactivity, children developed changes in their blood flow and arteries which would herald the start of serious cardiovascular problems in adult life.
- Fidgeting (i.e. playing, toying or jiggling) may counteract the negative effects of prolonged sitting. A recent study about fidgeting found that fidgeting may actually counteract the negative effects of sitting behind a desk all day [5]. The authors used data from the UK Women’s Cohort Study, following 12,778 women aged 37–78 years over a 12-year period. Women who were sitting for seven or more hours daily had a 30 % increased risk of dying from any cause, compared with those who sat less than 5 h a day.
- Seniors who work longer are healthier than those who are retired [6]. Researchers from the University of Miami examined data on more than 83,000 Americans, all of whom were at least 65 years old when they were interviewed; 13 % of them were still working part time or full time. Of those still working, 61 % held white-collar jobs. Compared with people with white-collar jobs, those who were unemployed or retired were almost three times more likely to report their health as ‘poor’ or ‘fair.’

## Nutrition

- Vitamin C may improve blood vessel tone in sedentary adults who are overweight or obese, as presented at the American Physiological Society’s annual meeting. Overweight and obese people who take a high dose of supplementary vitamin C daily may acquire cardiovascular benefits related to physical exercise. Subjects who took 500 mg of vitamin C daily had equal improvement in blood vessel tone as those who took up a three-month regimen of brisk walking five to seven times a week.
- Trans fatty acids (TFA) may not be harmful indicating that not all TFA are equal, and that several TFA might even be good [7]. The authors of the study assessed TFA levels in 3259 cardiac patients by measuring the fatty acid composition of erythrocyte membranes. During 10 years of follow-up, total TFA concentrations were associated with a reduced risk of cardiovascular death. The reduction in risk was mainly determined by naturally occurring TFA, especially trans-palmitoleic acid, which is rather specific for dairy products.
- Drinking coffee does not increase risk of atrial fibrillation [8]. Analysis of more than 76,000 participants in the prospective Cohort of Swedish Men and the Swedish Mammography Cohort showed that there was no association with coffee consumption in more than 7000 patients with atrial fibrillation.
- Carbonated drink consumption may be associated with higher risk of acute myocardial infarction outside of a hospital as presented at the 2015 European Society of Cardiology (ESC) Congress in London. The more money people spend on carbonated beverages the more likely they are to suffer heart attacks outside of a hospital. Meanwhile, expenditures on other beverages such as green tea, black tea, coffee, cocoa, fruit or vegetable juice, fermented milk beverage, milk and mineral water were not significantly related to out-of-hospital cardiac arrests.
- Honey, cane sugar, high-fructose corn sweetener have similar impacts on health. A study by the United States Department of Agriculture did find that the health effects of high-fructose corn sweetener and honey were essentially the same. It was found that daily doses of honey, cane sugar and high-fructose corn sweetener had equal impacts on blood sugar, insulin, body weight, cholesterol and blood pressure.
- A daily glass of red wine may help improve metabolic health in people with type-2 diabetes [9]. It was reported that one glass of red wine in the evening increased good cholesterol in diabetes patients by about 10 % compared with those who consumed white wine or water. The patients also showed fewer symptoms of metabolic syndrome such as hypertension, excess abdominal fat, high blood sugar and abnormal cholesterol levels.

## Smoking

Individuals who were exposed to second-hand smoke when they were children or when they were in the womb may have a higher risk of developing atrial fibrillation later in life. The findings were based on Data obtained from the Health eHeart Study, analysing 4976 participants, of whom 593 (11.9 %) showed atrial fibrillation [10].

## Stress

- Psychological distress in childhood may be associated with higher risk of heart disease and diabetes later in life [11]. Investigators studied 7000 people born in a single week in Great Britain in 1958 over the course of 45 years. It was found that the participants with persistent distress throughout their lives had the highest cardio-metabolic risk score when compared with those who reported low levels of distress throughout childhood and adulthood.
- A high-stress job may be linked to an increased risk for an ischaemic stroke [12]. The authors analysed data from six studies on high-stress jobs involving more than 138,000 participants who were followed for periods ranging from 3 to 17 years. It was found that people with high-stress jobs had a 22 % greater risk of stroke than people who worked in low-stress jobs. For women, the contrast was even more outspoken as women with high-stress jobs had a 33 % higher stroke risk compared with those in low-stress jobs.
- Contrary to stress, it was recently reported that cardiac patients with a positive outlook (‘sunny disposition’) are more likely to exercise, continue with their medications and to take other steps to protect against further cardiovascular complications. These findings were based on surveys and physical examinations of over 1000 adults with coronary artery disease [13].

## Sexual activity

Sexual activity does not trigger heart attacks or strokes [14]. In total, 536 cardiac patients between 30 and 70 years were questioned about their sexual activity before and after heart attacks and cerebral strokes. The authors found that < 50 % of men and < 30 % of women are getting information about sexual activity after heart attack from their physicians. It was concluded that sexual activity was not a risk factor for subsequent adverse cardiovascular and cerebrovascular events. It is therefore important to reassure patients that they need not be worried and should continue and/or resume their usual sexual activity.

## Acupuncture

Acupuncture may potentially benefit people with mildly or moderately elevated blood pressure [15]. Investigators found that blood pressure levels declined slightly in a small group of patients treated 30 min a week with ‘electro-acupuncture’—where the needles carry low-level electrical stimulation—at specific points of the body.

## Social media

- Four monthly text messages might stimulate health improvements for cardiac patients [16]. Receiving texts with motivating and informative messages led patients with coronary heart disease to make behavioural changes such as more exercise and less smoking. By the end of the six-month study it was shown that patients who had received the text messages had lower cholesterol levels, decreased blood pressure, and reduced body mass index.
- Interactive health tracking may help lower blood pressure in patients with hypertension, as presented at the American Heart Association Council on Hypertension Scientific Sessions 2015. Routine use of an interactive website or app that tracked health data and stimulated regular exercise and other healthy behaviours—structured as a game—was associated with significantly lower blood pressure among hypertensive participants. It was found that almost 50 % of study participants with high blood pressure (≥ 140/90 mmHg) at the start of the study showed a significant reduction in systolic blood pressure.

In summary, a healthy lifestyle remains a challenging world. The above-described new findings on lifestyle modification published during the past 6 months may have prominent effects on the occurrence of CVD. Nevertheless, cornerstones of lifestyle remain exercise, dietary influences, and smoking habits. European guidelines (version 2012) and systems such as HeartSCORE have been developed to assist physicians in advising patients [17, 18]. Yet, according to the EUROASPIRE IV study, a large majority of CVD patients do not achieve the guideline standards for secondary prevention with a high prevalence of persistent smoking, unhealthy diets, physical inactivity and consequently most patients are overweight or obese with a high prevalence of diabetes [19].

The present study by Achttien et al. [2] clearly shows the value of evidence-based clinical algorithms for exercise-based cardiac rehabilitation in patients with coronary artery disease and chronic heart failure according to their training goals. The proposed algorithms may serve to improve guideline adherence and the effectiveness of exercise-based cardiac rehabilitation. The findings were based on reviews of three Dutch guidelines and three European position statements. The current study elaborates on previous studies by Achttien et al. [20, 21] who showed the effectiveness of exercise-based cardiac rehabilitation during all phases of cardiac rehabilitation in patients with coronary artery disease and chronic heart failure. The implementation of these and other guidelines in clinical practice needs further evaluation as well as the maintenance of an active lifestyle after supervised rehabilitation. It is therefore important for all physicians and physician assistants to better implement these guidelines and to advise their patients on lifestyle both in primary and secondary intervention [22–25]. Worldwide attention to lifestyle modification in preventing CVD remains therefore paramount and crucial [26]. Currently, it is more than clear that both primary and secondary prevention by lifestyle modification are most effective in halting the progression of atherosclerosis and decreasing disease burden over a lifetime. The studies by Achttien et al. [2, 20, 21] are a valuable contribution to this field.
